# Differences of Perceived Image Generated through the Web Site: Empirical Evidence Obtained in Spanish Destinations

**DOI:** 10.3389/fpsyg.2016.01861

**Published:** 2016-11-24

**Authors:** Juan J. Blazquez-Resino, Ana I. Muro-Rodriguez, Israel R. Perez-Jimenez

**Affiliations:** ^1^Business Administration Department, University of Castilla-La ManchaToledo, Spain; ^2^Econometric Department, University of Castilla-La ManchaToledo, Spain

**Keywords:** perceived image, information search behavior, Web pages, destinations, Spain

## Abstract

In this paper, a study of the perceived destination image created by promotional Web Pages is expounded in an attempt to identify their differences as generators of destination image in the consumers' mind. Specifically, it seeks to analyse whether the web sites of different Spanish regions improve the image that consumers have of the destination, identifying their main dimensions and analysing its effect on satisfaction and intentions of the future behavior of potential visitors. To achieve these objectives and verify the hypotheses, a laboratory experiment was performed, where it was determined what changes are produced in the tourist's previous image after browsing the tourist webs of three different regions. Moreover, it analyses the differences in the effect of the perceived image on satisfaction and potential visitors' future behavioral intentions. The results obtained enable us to identify differences in the composition of the perceived image according to the destination, while confirming the significant effect of different perceived image dimensions regarding satisfaction. The results allow managers to gain a better understanding of the effectiveness of their sites from a consumer perspective as well as suggestions to follow in order to achieve greater efficiency in their communication actions in order to improve the motivation of visitors to go to the destination.

## Introduction

Over recent decades, the tourism industry has experienced continuous growth and has become one of the main economic sectors in the world. Different studies have indicated the strategic role of the tourism industry on the nation's economic growth (Sarıışık et al., 2011) and as a principal driving force for new destinations development (Kim et al., 2013). In Spain, as one of the most important tourist destinations in the world, the tourism industry has had a considerable influence on the national economy, which has become a strategic sector.

However, tourism marketers must face an increasingly complex, competitive, and saturated marketplace overall (Echtner and Ritchie, 1993). Recently the effects of the Global Economic Crisis have been added. Although the tourism industry has also proven to be one of the strongest sectors during the Crisis, it has had a fundamental impact on tourist's demand. Therefore, this context creates the need to redesign the management strategies of the destinations (Sirgy and Su, 2000). In general, many destinations focus their strategies on the confidence that visitors will be attracted by the destination's tangible resources, forgetting that success is not in possession but in its effective deployment (Ritchie and Crouch, 2000). Thus, destinations with limited tangible resources, but managed efficiently, can make the visitor's perception better than destinations with more valuable resources but unable to achieve an effective value proposal (McCartney et al., 2008). Destination marketing organizations (DMOs) make substantial efforts to establish positive destination image because it is important in the process of attracting potential visitors (Fakeye and Crompton, 1991; Sirgy and Su, 2000). The assessment of the destination image can assist managers by identifying the strengths and weaknesses of their destination, helping predict tourists' behavioral intentions (Fakeye and Crompton, 1991; Bigne et al., 2001). In particular, potential visitors with limited knowledge of destinations depend on their perceived image of a destination when it comes to making choices (Um and Crompton, 1992; Beerli and Martín, 2004). The literature clearly shows that the creation and communication of an image for a destination represent a true competitive advantage and an effective strategy for competing in the market (Gallarza et al., 2002; San Martín and Del Bosque, 2008).

To create a favorable perceived image it is essential to analyse how the process of promotional destination information is developed by the visitor. Previous studies about consumer behavior have tried to understand how the operating the processing of information is, what variables can become crucial and which formats, means, or arguments are the most persuasive (Rodríguez-Molina et al., 2015). Girard and Gartner (1993) stated that best way to appreciate a destination is to visit it. However, when consumers have not previously visited the destination, they face a series of conditions of uncertainty whose main cause is the lack of objective criteria for evaluating the destination. To reduce this uncertainty, visitants used different information sources, both internal, and external, trying to acquire as much information as possible to form a perceived image about the destination prior to the visit.

In this sense, the Internet is becoming one of the most important ways of collecting tourism information and creating a perception of the destinations image. The development of the Internet as a means of communication has changed the behavior of external information search by potential visitors to the destination. As information it is meant that, “*the Internet constitutes a communication channel that many traditional information sources leverage*” (Llodra-Riera et al., 2015, p. 319). From the destinations perspective, the Internet offers numerous advantages over traditional communication sources. A Web page is a dynamic and interactive source of information, rich in content (Pan and Fesenmaier, 2006), which can generate virtual experiences through environmental simulations (e.g., Simulation of real visits). Thus, a destination web page becomes a fundamental instrument in promoting the destination and it leads to a strong feeling of destination image in the visitor (Cho et al., 2002).

Previous research has focused both on understanding how visitors use online communication channels to search for information, as in the way in which the DMOs can be used to improve their promotion strategies (e.g., Pan and Fesenmaier, 2006; Buhalis and Law, 2008; Papathanassis and Knolle, 2011; Ho et al., 2012). However, although a relative abundance of studies have focused on the effect of promotional materials on the destination image (e.g., Gartner, 1989; Sonmez and Sirakaya, 2002), few researchers have focused on the effect of the Internet. Most studies have focused on the analysis of the Internet and on evaluating the performance of websites in terms of content and accessibility, using content analysis of online platforms or analysing user generated content from the experiences of visitors (e.g., Leung et al., 2011; Llodra-Riera et al., 2015; Sun et al., 2015; Tseng et al., 2015). As sources of information, destination websites have reached an important effect on image formation processes (Choi et al., 2007), despite this, few researchers have empirically examined the role of website information on the potential visitant's destination image (e.g., Lepp et al., 2011; Jeong et al., 2012). This study aims to complement the previous research.

The objective of the research was to identify the effect of travel websites on potential tourists' images. Specifically, we seek to analyse whether the information provided by the web pages of different DMOs causes the previous destination image could be significantly different after exposure. In addition, it aims to determine the relationship of the image generated on satisfaction with the tourism destination and on future intentions to recommend or visit. To reach the research objectives the websites of different Spanish tourism destinations have been taken into consideration. Using the findings obtained, we will draw a set of academic and professional implications which enable the development of more efficient online communication strategies.

## Literature review

### Information search behaviour in tourism

The way information is processed will influence the consumers purchase decision (Frias et al., 2008). For that reason, for a long time, research in consumer behavior has focused on analysing how the consumer process of information is. In an actual dynamic environment, the need to better understand how consumers acquire knowledge, and search information is important for marketing management decisions and service delivery (Gursoy and McCleary, 2004). Information search is defined as “*the motivated activation of knowledge stored in memory or acquisition of information from the environment*” (Engel et al., 2001, p. 106).

The main purpose of the information search is to support the process of decision making, reduce risk and uncertainty, and product choice. Hirschman and Wallenoorf (1982), stated that consumers engage in information search for these basic reasons: enhancing the knowledge about product or services and alternatives; and reducing the risk of incorrect choices and future purchase decisions. Risk reduction is considered particularly crucial in non-routinized and extensive decisions regarding acquirement of expensive and complex products, and when people often are strongly involved in decision-making processes. In this sense, most researches stated that tourists develop very extensive search of information due to the characteristics of tourism products, it cannot be tried before purchasing. Therefore, to reduce the perceived risk in consumption of unfamiliar tourist products, travelers often use multiple sources of information before making a final decision. Information search does not guarantee satisfaction in consumption experiences but help to reduce visitors perceived risks and therefore, optimize their decisions (Leung et al., 2011).

Fodness and Murray (1998) identify three dimensions for information search in any given purchase situation:

1. The *spatial dimension* indicates the locus of search activities. It is possible to distinguish between internal search, by retrieving memories; or external search, obtaining information from market-related sources.
2. The *temporal dimension* reflects the timing. There is an ongoing search, which lets you create a “*knowledge base*,” and pre-purchase search, developed to face a particular problem of purchase.
3. Finally, the *operational dimension* considers the search behavior. It focuses on sources used and their relative utility for decision-making.

The traveler's search of information is one of the most frequently examined topics by tourism researchers (Schul and Crompton, 1983; Fodness and Murray, 1997, 1998; Vogt and Fesenmaier, 1998; Gursoy and McCleary, 2004; Bargeman and van der Poel, 2006; Hyde, 2008), and all decision-making models include pre-purchase hunt for information as key components (e.g., Howard and Sheth, 1969; Schmidt and Spreng, 1996; Engel et al., 2001). For tourism destinations, information search is one of the first steps of the vacation decision-making process and has influence on travel behaviors, such as where to go, how long to stay and how much to spend (Romf et al., 2005).

Whenever a visitor realizes that they need to make a decision, initially an information search takes place internally as the basis for making a vacation decision. Internal sources include previous experiences, with the destination or similar, and the knowledge accumulated through an ongoing search process (Fodness and Murray, 1997; Vogt and Fesenmaier, 1998). However, if internal information proves inadequate or not up-to-date, travelers are likely to use additional information from external sources. In most travel decisions, the search is predominantly external, particularly for new destinations, representing a wide variety of sources of information, and considerable time (Fodness and Murray, 1997).

An important question of practical importance is where tourists obtain external travel-related information. External search consists not only in collecting information from the marketplace but also from a variety of more or less independent or unbiased sources such as news media, guidebooks, and acquaintances. Visitors tend to use a broad combination of external information sources as their search strategies. Different researchers (e.g., Fodness and Murray, 1997; Vogt and Fesenmaier, 1998; Gursoy and Umbreit, 2004) have categorized external information sources as: (1) social, personal, marketing, and editorial; (2) commercial and non-commercial; (3) marketer controlled, reseller information, thirdparty independent organizations, interpersonal sources, and direct inspection; and (4) consumer dominated, marketer dominated, and neutral sources. Travelers rely on both marketing-dominated (mass media, travel brochures, guidebooks) and non-marketing-dominated (includes friends, relatives, and personal experiences) sources of information for finding information related to travel and plan the trips.

The visitor's search of information will be as varied and long as the benefits of acquiring information is higher than the costs (Gursoy and McCleary, 2004). Not only monetary costs but also the time spent can influence on the external search. In this sense, the Internet becomes the indispensable channel for people seeking to use tourism information, also in planning and purchasing a travel (Buhalis and Law, 2008). The advantages of Internet as an information source include, first of all, interactivity, but also customized information, low cost, wide coverage, and comprehensive functions (Ho et al., 2012). On the other hand, with the huge amount of information available to travelers, the Internet constitutes an important platform for information exchange between consumer and industry suppliers.

From a consumer behavior perspective, the Internet has gained considerable importance as a communicative means of sharing and disseminating information, different from mass media (television, radio, newspaper, or magazine), becoming one of the main sources of tourist information (Li et al., 2009). The unique characteristics are affecting the consumer behavior (Dholakia and Bagozzi, 2001). The Internet is partly used for practical pre-departure purposes such as travel planning, booking, and payment of tourism products (Hyde, 2008). The Internet offers a rich environment for the information needed by potential travelers who want to gain familiarity with a destination and to locate something of interest to them (Ho et al., 2012).

The research related to the Internet tourism information search has attracted much attention. Earlier studies focused on information search behavior, that utilizes the Internet and what kind of tourism information they are looking for (Pan and Fesenmaier, 2006; Xiang et al., 2008). However, recent researches have focused on the progress of tourism information search behavior caused by the changes of information technology, as social media or blogs, more related with informal information, such as the travel experiences and recommendations of travelers (Xiang and Gretzel, 2010; Sun et al., 2015).

In this way, the Internet constitutes the most powerful communication tool for reaching and attracting more tourists due to its interactive capacity (Beldona and Cai, 2006). For this reason, many tourist destinations have decided to use Web pages as a means of promotion. The Website of a tourist destination is the center from which its attractions spread out to net browsers and other members of the value chain, such as hotels and restaurants. Despite this, given the competition, it is more and more difficult to stand out in this cyber space. Survival depends on different factors related to the design of the Web (Mayordomo, 2003), such as the functionality (content) and usability (ease of use) of the pages. The majority of the Webs of tourist destinations focus on usability and on providing information, since they consider it sufficient to attract potential tourists (Zach et al., 2007). However, a Web page must be a balance between visual and graphic design, business logic, and practical utility. The destination Websites serve as a calling card and they have to be sufficiently attractive for the tourist to decide to visit that destination. The content of destination web pages is especially important because it directly affects the perceived image of the destination, creating virtual experiences that increase the more interactivity is present (Doolin et al., 2002 p. 557). Therefore, the structure and content of the Web must take into account the following questions: what destination image are we seeking to transmit? How can added value be provided to the potential tourist in order to improve the overall image? (Mayordomo, 2003).

### Perceived image of tourism destination

Perceived destination image has been widely studied in literature. Nowadays, there is a general consensus that the destination image has a key influence on the visitor's travel decision, consumer's satisfaction and destination evaluation (Bigne et al., 2001; Gallarza et al., 2002; Beerli and Martín, 2004; Chen and Tsai, 2007). In general, visitors have a limited knowledge of the destinations which they have yet to visit, thus bestowing an important role on image when it comes to attracting visitors (Huang and Gross, 2010). Destinations with strong, positive and recognizable images will become more probable to be included in the visitor's process of decision-making (Echtner and Ritchie, 1993; Beerli and Martín, 2004). Baloglu and McCleary (1999) postulated that perceived image is a key indicator of destination performance and of the visitor's satisfaction, hence, influencing travel behavior, potential travel intention, and consumption patterns. Thus, the perceived image is one of the most important aspects in the positioning of a destination (Echtner and Ritchie, 1993) since it contributes to creating factors that distinguish it from the competition (Li and Vogelsong, 2006).

Tourism scholars have come up with numerous definitions of destination image, nearly as many definitions of the image as researchers studying it (Gallarza et al., 2002). Although some authors identify it as the perception or set of impressions regarding the place (Phelps, 1986; Fakeye and Crompton, 1991), most consider that the perceived image is the mental representation of a destination (Alhemoud and Armstrong, 1996) or an attitudinal concept consisting on the sum of beliefs, ideas and sensations which individuals hold of a place or destination (Crompton, 1979; Baloglu and McCleary, 1999; Bigne et al., 2001; Li et al., 2009).

Recently, Jani (2016) indicated there is not any accurate definition of perceived destination image due to the presence of different destination image components. However, in general, the destination image is composed of two main dimensions: cognitive and affective (Gartner, 1993; Baloglu and McCleary, 1999; Beerli and Martín, 2004). For instance, Echtner and Ritchie (1993) stated that the destination image vary on a continuum from functional destination attributes, structural elements, or physical characteristics of destination easily observable or measurable, to psychological characteristics, abstract and not easily observed. While cognitive image is based on the perceptions of structural or tangible physical elements, the affective dimension reflects a psychological response, the tourists' emotion or feeling about the destination (Echtner and Ritchie, 1993; Baloglu and McCleary, 1999; Beerli and Martín, 2004). Cognitive components allow us to understand the process of choosing a tourist destination (e.g., Chen and Hsu, 2000). In this sense, MacKay and Fesenmaier (1997:539) claim that “*destination image is composed of different products or attractions and attributes which create an overall impression*.” This means that destination image is determined by the notion that the tourist has regarding the attributes which comprise it and whose presence or absence determines the tourist's perception. Different research has found a cognitive dimension as ideal destination images (Chen and Hsu, 2000; Sonmez and Sirakaya, 2002; Bonn et al., 2005; Barroso et al., 2007). However, affective image also influences on the evaluation of destination image (see Nghiêm-Phú, 2014 for review) and therefore influences the decision-making and the desire to visit a destination.

On the other hand, the different categorization of destination images implies that the concept is liable to be defined in various ways using different stages of travel and different sources of travel information (Gartner, 1993; Beerli and Martín, 2004). Thus, the Gunn (1988) stated perceived image can be modified following a sequence: accumulation of mental images of destination, modification of the images through information, and modification of the destination image after experiencing the destination. Then, the perceived destination image includes organic, induced, and complex or modified image (Jeong et al., 2012). The information originates in numerous and diverse sources (Gartner, 1993). Firstly, an organic image is created through general life experiences and non-commercial accumulated information sources such as movies, newspapers, periodicals, books, and personal sources, while induced images are created by commercial travel information sources such as the tourism promotion literature, including magazine articles, guidebooks, Web pages, and TV promotions (Echtner and Ritchie, 1993).

Various researchers have further developed Gunn (1988) concept of image change (Fakeye and Crompton, 1991; Gartner, 1993; Baloglu and McCleary, 1999; Beerli and Martín, 2004; Yuksel and Akgul, 2006). Based on the effect of travel information search, Lin et al. (2007) categorized destination image into baseline image, held before collecting travel information, and enhanced image after having collected travel information. Phelps (1986) categorized destination images into primary and secondary depending on the information sources used. While primary images are formed through internal information such as past experiences, secondary images are influenced by information received from some external sources. Secondary sources fulfill three basic functions in destination choice: to minimize the risk of making a wrong decision, to create destinations image, and to justify a subsequent choice (Mansfeld, 1992). Fakeye and Crompton (1991) used the term “*evolution image*” to explain the process of image change, adding the additional step, the complex image that is generated from the actual visitation of a destination. In this sense, from organic to induced reflect pre-visit image and complex image represent the post-visit image, after experiencing the destination.

The information sources used by the future visitor are one of the factors widely regarded for its influence on the generation of the pre-visit destination image (Frias et al., 2008). In this sense, it becomes very important to study whether the image projected by the destination promotional materials correspond to those held by visitors (Stabler, 1987). Tasci and Gartner (2007, p.403) claim that to achieve success of a tourist destination is very important to have an adequate image development. Although it is the subjective assessment of external stimuli that forms the image of the destination (Gartner, 1993), it becomes very important to consider what stimuli wants to be present in the DMOs, because sometimes the projected image might not be the same as the received one. In fact, the information transmitted between suppliers, intermediaries and recipients has become more complicated with the arrival of the Internet (Choi et al., 2007).

With the increasing popularity of the Internet, DMOs have used official travel websites as main communication channels (So and Morrison, 2003; Kaplanidou and Vogt, 2006). Due to the multiple dimensions of the destination image and the complexity that the Internet has brought, it is important to examine the provided information to understand the process of image formation in the online context. The intangible characteristics of destination product means that the transmission of the most important images of the destination can only be carried out through their representation in the graphic media and/or audiovisual means. The Internet has great potential to influence on consumers' perceived images it allows to generate virtual experiences (Gretzel et al., 2000) and present a virtual image of the destination that might reduce the perceived risk of a wrong choice.

Previous online information studies have shown two major trends. Some researchers about the travel websites have focused on analysing the operation of the website in terms of accessibility and content (e.g., Bai et al., 2008; Tang and Jang, 2008; Loda et al., 2009; Woodside et al., 2011; Rodríguez-Molina et al., 2015). Their results have shown that website design and Internet marketing features contribute to an effective delivery of messages, quality of products and services, and brand image. Other studies have focused on how the websites influence on the formation of the pre-visit destination image travel intentions. Nevertheless, there are only a few studies (e.g., Frias et al., 2008; Lepp et al., 2011; Jeong et al., 2012; Rodríguez-Molina et al., 2015) and the results are inconclusive. For example, Lepp et al. (2011) found that exposure to the website improves the image and reduces the perceived risk of the destination of Uganda. Jeong et al. (2012) revealed that exposure to a travel website significantly affected most cognitive and overall destination images. Other studies demonstrate that the vast quantity of information available on the Internet leads to information overload and disorientation among its users (Frias et al., 2008; Rodríguez-Molina et al., 2015).

## Procedure

It is considered that the perceived destination image is multiple, dynamic and complex (Gallarza et al., 2002). Previous literature asserts that image-building is related with the unique identity of a destination (Park and Petrick, 2006). Given that not all the destinations possess the same resources nor combine the same characteristics, the final configuration of the image will be conditioned by the destination itself. Furthermore, not all tourists will identify the resources of the destination in the same way. Therefore, as the first objective of the research, we propose to delimit the main aspects which determine destination image. To achieve this objective, we posit the following hypothesis:

***H1. Configuration of the perceived image varies according to the tourist destination***.

In literature it is possible to distinguish different main conceptual trends about the effect of information sources on the creation of image (Baloglu and McCleary, 1999; Beerli and Martín, 2004; Huang and Gross, 2010). The cognitive-affective model is the one most used when it comes to studying the relationship between destination image and other constructs of interest. Crompton (1979) was the first author to speak of the cognitive image which reflects the idea that a person has regarding the physical properties of a place. Some authors (Echtner and Ritchie, 1993; Walmsley and Young, 1998) claim that image is not only cognitive (beliefs), but that it also possesses an affective component (feelings), which comprises the overall impression, called total image (Baloglu and McCleary, 1999), which produces the positive or negative evaluation of the destination (Beerli and Martín, 2004).

In this regard, some works have identified the significant influence of information sources on the cognitive image, but not on the affective one (Baloglu and McCleary, 1999). However, the printed information media, such as brochures, are limited when it comes to sending messages which transmit emotions, unlike the online media whose dynamic nature, and interactivity enable them to offer information related to affective aspects more effectively (Kim and Fesenmaier, 2008). In a more recent study, Li et al. (2009) conclude that the affective and overall image change after browsing the Web, while the cognitive one remains stable, greater stimuli being necessary to generate significant changes in the cognitive image. Following the latter observation, it is important to carry out new studies in order to determine the effect of tourist Web Pages on the image of the tourist by studying whether the previous image, both cognitive and affective, differs from that shown after browsing the Web. Regarding this aspect, we posit the following research hypotheses:

***H2. Tourist Web Pages modify the tourists' total image regarding to the destination***.
  ***H2a. Tourist Web Pages modify the tourists' cognitive image regarding the destination***.
  ***H2b. Tourist Web Pages modify the tourists' affective image regarding the destination***.

On the other hand, destination image not only affects the destination choice but also its assessment and the future behavior of the tourist (Bigne et al., 2001; Chen and Tsai, 2007; Chi and Qu, 2008). Satisfaction is a key factor in the success of tourist consideration, given that it is linked to the choice of destination, the consumption of products and services and also the decision to return to the destination. The previous image significantly determines the expectations of the tourist toward the destination, generated by the information search processes, something which will affect the satisfaction attained by the tourist. The analysis of the effect of image on satisfaction with the destination enables us to identify the key attributes which ensure that the destination can reach or surpass the expectations of the customer and, therefore, ensure a return to the destination. Bearing in mind this approach, and considering tourist satisfaction to reflect the perceived image, in the present paper we consider that the relationship between destination image and the behavioral intention of the tourist is intermediated by satisfaction with the destination after the information search. In consequence, we posit the following hypotheses:

***H3. Perceived image positively affects tourist satisfaction with the search for online information***.
***H4. Satisfaction with the search for online information regarding a tourist destination positively affects future behavioral intentions***.

To attain the objectives proposed and verify the hypotheses posited, a procedure is designed for collecting the information based on a laboratory study, due to the need of establishing an environment in which the researcher can control other external variables which might affect the sample units. The main features of the research are presented in Table 1. To best knowledge, only few studies used experimental design to analysis the effect of internet-based information on the tourist image change (e.g., Lepp et al., 2011; Jeong et al., 2012).

**Table 1:** **Technical data**.

Universe	Spanish tourists who use the Internet
Analysis technique	Laboratory study
Sample size	177
Sample design	Convenience sample


To obtain the necessary information, a personal structured questionnaire is given to each sample unit with instructions for its correct completion. In the first place, those participating must respond to a set of questions which seek to find out the prior image they have of a specific destination. Subsequently, they must work out an information search process for a future trip in the official web page of the same destination, carrying out a totally free browse. Once the search is performed, the participants in the survey must respond again to questions regarding the destination image after exposure to the website information, as well as their satisfaction and future intentions to make a visit. Each group is exposed to one of the tourist web pages included in the study.

To perform the analysis of the differences in the composition of the destination image, it was necessary to select the webs of different tourist destinations. Due to the fact that the final delimitation of the population of the study was national tourists, the choice of destinations is performed by using the data obtained by the survey: *Spanish Tourist Movements—Familitur*. The choice was based on criteria related to the total number of visits and differences in the tourist products and services offered. The final choice corresponds to the Autonomous Communities of Castilla-La Mancha, Castilla-Leon and Andalusia.

In the development of the measurements of each of the variables included in the study, we have followed the recommendations established in the literature regarding the determination of the nature of the constructs to avoid committing an incorrect specification (MacKenzie et al., 2005). Most studies about perceived image have used a structured approach, employing Likert scale and/or semantic differential to measure the cognitive and affective dimensions (e.g., Chen and Hsu, 2000). Typically, researchers grouped a set of pre-determined image attributes, based on literature review and study context, into several dimensions by data reduction techniques (Bigne et al., 2009). Although Gallarza et al. (2002) showed a list of the most used elements, there is not a battery of image items widely accepted and applicable to different types of destinations.

In the development of the marketing measurements, in general, the “almost automatic acceptance” of the models of latent and reflective constructs has occurred (Diamantopoulos and Winklhofer, 2001: p. 274), which establish that the causality flows from the latent construct toward the observable measurements, which are manifestations of the construct. However, some authors claim that in certain cases the relationship between the latent variable and the observable variables is the opposite (Edwards and Bagozzi, 2000), giving rise to models of formative constructs, which mean that the measurements form the construct. In this respect, the destination image is identified as a formative construct, where the image of each of the attributes of the destination forms the overall image. Currently, other constructs related to attitude are being analyzed using formative construct models (Zabkar et al., 2010; Blazquez-Resino et al., 2015). Even though the literature has paid less attention to the development of formative measurements, some previous studies (MacKenzie et al., 2005; Petter et al., 2007) have enabled the identification of a series of stages to be followed in the development of formative measurements (See Table 2).

**Table 2:** **Development of the formative measurement of the perceived image**.

	**Theoretical framework**	**Application**
**Identification of the concept**	• Conceptual domain	Mackay and Fesenmaier (1997, p. 539): a tourist's image of a specific destination is defined by the attributes, resources and capacities which characterize said destination. Chin et al. (2008): determination of the measurement should be based not only on the way of measuring the variables but it is also necessary to analyze how the elements are created.
	• Dimensionality	Cognitive dimension (21 variables) - Natural resources (3) - Infrastructures (2) - Hotel services (3) - Leisure (3) - Culture, history and art (4) - Political-economic factors (2) - Environment (3) - Social aspects (1)
		Affective dimension (6 variables)
**Concept validation**	• Content validation
	♦ Review of the literature	Sonmez and Sirakaya, 2002; Beerli and Martín, 2004; Lin et al., 2007; Huang and Gross, 2010
	♦ Q-Sorting	23 persons outside the research

The final scale is composed of 21 variables which measure the cognitive dimension, grouped into 8 dimensions 6 variables which are grouped into the affective dimension. Respondents must “*give the opinion about the following tourism destination characteristics*,” based on the 29 variables included in the analysis. A seven-point *Likert* scale (from: *Totally Disagree* to *Totally Agree*) was used for the cognitive dimension, while a seven-point *Semantic Differential Scale* for the affective one.

For the development of the measurements of satisfaction and behavioral intentions the classical model is followed (Churchill, 1979), carrying out a broad review of the literature related to each of the constructs with the aim of recognizing those scales and measurement variables which have been verified and validated in previous studies. Satisfaction is measured by means of three questions (“*It's a worthy destination for visiting*,” “*I like as a tourist destination*” and “*My overall perception of the destination is very good*”) with which it is sought to estimate the overall destination evaluation obtained from the information search in the web site. Future behavioral intentions are studied by means of the intention to visit the destination in the future (“*I will try to go to the destination in the coming years*” and “*I think that I will visit the destination in the future*”) and intention to recommend with two questions (“*I will recommend the destination*,” “*Encourage family and friends to visit the destination*”). For these variables, a seven-point *Likert* scale is used, from “Totally Disagree” to “Totally Agree.”

## Results and discussion

First, the descriptive results show how the information obtained through the website of the destination influences the previous ratings (see Table 3). Although in most of the analyzed variables exceeds the value given after browsing the website, for some variables and destinations the effect is negative.

**Table 3:** **Previous vs. after evaluation—means values**.

**Factor**	**Indicator**	**CASTILLA-LA MANCHA 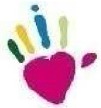 **		**Andalusia 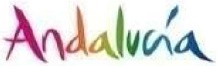 **		**CASTILLA Y LEON 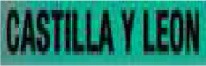 **	
		**Previous**	**After**	**Previous**	**After**	**Previous**	**After**
NAT.RES	Climate	5.14	5.37	5.31	5.71	4.39	4.88
	Flora and fauna	5.02	5.61	4.76	5.44	5.24	5.39
	Parks and nature zones	5.47	5.88	5.14	5.53	5.17	5.46
INFR	Roads and access ways	3.54	3.80	4.49	4.68	4.32	4.51
	Public transport	3.83	4.02	4.15	4.37	4.08	4.47
HOT.SER	Hotel infrastructures	4.59	5.25	5.08	5.51	4.69	5.15
	Restaurants	5.19	5.49	5.03	5.66	5.08	5.24
	Tourist information	4.39	5.44	4.88	5.27	4.56	4.41
LEIS	Night life	5.07	4.59	5.63	5.73	4.54	4.63
	Entertainment and leisure	5.05	5.07	5.51	5.73	4.68	4.83
	Shopping establishments	4.90	4.56	4.95	5.08	4.39	4.49
CULT	Gastronomy	5.88	5.8	5.42	5.61	5.54	5.58
	Cultural activities	4.95	5.75	5.1	5.76	5.19	5.37
	Customs	5.51	5.69	5.46	5.69	5.25	5.31
	History	5.98	6.03	5.37	5.71	5.39	5.71
POL.ECO	Prices	4.66	4.75	4.15	4.39	4.34	4.25
	Security	5.32	5.34	4.42	4.58	4.78	4.76
ENVI	Environment	5.07	5.61	5.54	5.78	5.15	5.34
	Cleanliness	4.64	5.19	4.34	4.69	5	5.19
	Traffic	4.05	4.61	3.9	4.19	4.36	4.71
SOCI	Hospitality	4.76	5.02	5.44	5.39	4.73	4.9
AFFE	Unattractive-very attractive	4.37	4.9	5.66	5.81	4.64	4.36
	Boring-fun	4.42	5.27	5.71	5.9	4.37	4.85
	Unpleasant-pleasant	5.2	5.59	5.56	5.92	5.1	5.29
	Stressful-relaxing	5.14	5.68	4.9	5.22	5.37	5.42
	Conventional-exotic	3.34	3.81	4.36	4.73	3.86	3.69
	Old-modern	3.41	3.68	4.49	4.75	3.63	3.49

In this sense, for the contrast of the hypothesis, the structural equation modeling method (SEM) is used. It is possible to find two different types of modeling: SEM based on co-variances and SEM based on components, also called Partial Least Squares Path Modelling (PLS PM). Although the first is the most used, PLS PM is an especially useful technique for incorporating formative constructs into the structural model (Petter et al., 2007). Therefore, PLS PM is considered to adapt to the proposal of the current research, performing the analyses by means of the SmartPLS 2.0 statistical pack.

PLS PM, as an SEM analysis model, is determined by two fundamental elements: estimation of the measurement model, where the relationship between the indicators and the latent construct is determined, and an estimation of the structural model, where the relationships between the constructs are evaluated by means of the path coefficients and their significance. Therefore, as a prior step to verifying the hypotheses posited, it is necessary to analyse the psychometric properties of the measurement model, distinguishing between the reflective and formative measurements, given that the estimation and validation framework is different.

To analyse the reflective measurement in PLS, the study of their reliability, convergent validity and discriminant validity is carried out (Gefen and Straub, 2005). The reliability of the constructs is examined by means of Cronbach's alpha and the compound reliability, automatically provided by the SmartPLS 2.0 program. The results obtained in both tests show values which exceed the recommended threshold of 0.7 (Churchill, 1979) and even the stricter one of 0.8 (Nunnally, 1978), which confirms the consistency and reliability of the constructs used. Convergent validity is analyzed by means of the study of the sizes of the factorial loads and the Average Variance Extracted, which indicates the variance captured by a factor with respect to the variance due to the measurement error. In the first case, the results show that the factorial loads of the measurement variables on their respective constructs exceed the minimum value of 0.7 (Chin, 1998), all the values being significant. On the other hand, the convergent validity is adjusted when the value of the Variance Extracted is >0.5, a value which is considerably exceeded given that all the values are close to or >0.8.

Finally, the discriminant validity is analyzed by means of the cross-loading analysis between the indicators and the constructs, where all the loads of the indicators on their latent variable must be greater than the loads on the rest of the factors. Secondly, the Fornell and Larcker (1981) is applied, which establishes that the variance shared between two constructs, measured by the square of their cross loading, must be lower than the Average Variance Extracted of any of the constructs. The results of both tests enable us to confirm the discriminant validity of the reflective constructs.

On the other hand, analysis of the reliability and validity of the formative indicators is carried out by means of the analysis of the weights of the elements on their corresponding formative constructs (Chin, 1998) and their respective significance (Petter et al., 2007). The weights reflect the contribution of the individual indicator on the construct. In PLS, the significance can only be estimated by the re-sampling method using bootstrapping techniques^1^. Following the recommendation of Brown and Chin (2004), 500 subsamples are generated of the same size as the original sample where it is necessary for the loads to be significant to at least 0.05 (Gefen and Straub, 2005).

The results obtained (See Table 4) allow us to accept Hypothesis 1, which establishes that the final configuration of the image depends on the specific destination analyzed. In the present study it can be observed how each of the dimensions of the image is determined by a number of different variables according to the tourist destination.

**Table 4:** **Analysis of the validity of the image measurements**.

**Factor**	**Indicator**	**CASTILLA-LA MANCHA 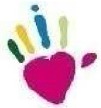 **		**Andalusia 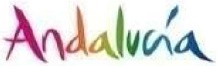 **		**CASTILLA Y LEON 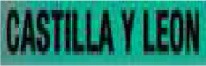 **	
		**Weight**	***t*-value (bootstrap)**	**Weight**	***t*-Value (bootstrap)**	**Weight**	***t*-value (bootstrap)**
NAT.RES	Climate	0.357	1.520	0.606^***^	2.586	0.224	0.854
	Flora and fauna	−0.372	0.959	−0.054	0.171	0.702^*^	1.955
	Parks and nature zones	1.100^***^	4.563	0.626^**^	2.049	0.199	0.490
INFR	Roads and access ways	0.226	0.552	0.796	1.354	1.242^***^	5.889
	Public transport	0.825^**^	2.340	0.268	0.412	−0.397	1.118
HOT.SER	Hotel infrastructures	0.300	0.848	1.089^***^	7.024	0.406^**^	2.073
	Restaurants	0.334	1.110	0.275	1.468	0.291	1.296
	Tourist information	0.536^**^	2.236	−0.384	1.133	0.621^***^	2.975
LEIS	Night life	0.098	0.401	0.126	0.243	0.299	1.034
	Entertainment and leisure	0.580^***^	2.609	1.021^***^	3.126	0.530^**^	2.274
	Shopping establishments	0.524^***^	2.831	−0.106	0.439	0.316^*^	1.935
CULT	Gastronomy	0.456^**^	2.244	−0.048	0.237	0.184	0.951
	Cultural activities	0.204	1.130	0.349^*^	1.732	0.495^**^	2.393
	Customs	0.315	1.369	0.607^**^	2.914	0.395^**^	2.451
	History	0.296^*^	1.960	0.277	1.340	0.126	0.510
POL.ECO	Prices	0.965^***^	5.892	−0.176	0.364	0.337	1.293
	Security	0.092	0.313	1.059^**^	2.385	0.887^***^	4.544
ENVI	Environment	1.028^***^	3.514	0.703^***^	3.541	1.053^***^	13.852
	Cleanliness	−0.036	0.098	0.533^***^	1.976	−0.129	0.917
	Traffic	−0.289	0.886	0.155	0.793	−0.031	0.179
SOCI	Hospitality	1	0	1	0	1	0
AFFE	Unattractive-very attractive	0.356	1.460	0.191	0.560	−0.022	0.100
	Boring-fun	0.088	0.262	0.643	1.590	0.552^***^	2.684
	Unpleasant-pleasant	0.716^***^	3.577	−0.479	1.055	0.440^**^	2.360
	Stressful-relaxing	−0.170	0.9190	0.215	0.938	−0.096	0.644
	Conventional-exotic	−0.224	1.082	0.793^***^	3.153	0.077	0.520
	Old-modern	0.570^***^	2.741	−0.181	0.781	0.213	1.203

*** *p < 0.01*;

** *p < 0.05*;

* *p < 0.10*.

*NAT.RES, Natural Resources; INFR, Infrastructures; HOT.SER, Hotel services; LEIS, Leisure; CULT; Culture, history and art; POL.ECO, Political-economic factors; ENVI, Environment; SOCI, Social aspects; AFFE, Affective dimension*.

For the verification of the rest of the hypotheses, it is necessary to perform the analysis of the structural model, which will enable an estimation of the path coefficients and their significance. In Table 4, we can observe the results of the structural analysis by means of the PLS, showing the structural coefficients, which indicate the strength of the relationships between the variables. For the analysis of the stability and significance of the parameters, which allows us to support the relationships established in the hypotheses, the bootstrapping technique generating 500 subsamples with the same size as the original sample was used. In the same way, the validity of the model is verified by means of analysis of the *R*^2^ value, which measures the predictive power, following the criteria proposed by Falk and Miller (1992) that the *R*^2^ of each of the dependant constructs must exceed the value of 0.1.

The third hypothesis establishes a positive effect of the dimensions which determine the image of a destination through online promotion on tourist satisfaction. From the results obtained (See Table 5), it is not possible to reject the hypothesis completely, given that it can be observed that the influence of the image generated on tourist satisfaction is different per the tourist destination analyzed. For the three destinations analyzed, the affective image exercises a significant influence on satisfaction. However, the dimensions of the cognitive image show distinct effects according to the destination. Specifically, in the Castilla-La Mancha Web, satisfaction is significantly influenced by the Cultural, Historical and Artistic dimensions (π = 0.4483, *p* < 0.01) and Leisure (π = 0.219, *p* < 0.05). For Andalusia satisfaction is determined by the Natural Resources (π = 0.0994, *p* < 0.05), Hotel Services (π = 0.0666, *p* < 0.10) and the Culture, History and Art (π = 0.219, *p* < 0.05), while the Natural Environment (π = 0.2267, *p* < 0.05) is the only cognitive dimension which attains a significant value over satisfaction in Castilla-Leon.

**Table 5:** **Structural relationships between image and satisfaction**.

**Relationship**	**CASTILLA-LA MANCHA 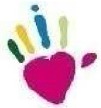 **		**Andalusia 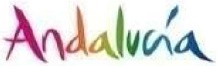 **		**CASTILLA Y LEON 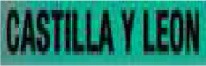 **	
	**β Standard**	***t*-Value (*bootst*)**	**β Standard**	***t*-value (*bootst*)**	**β Standard**	***t*-value (*bootst*)**
NAT.RES → SAT	0.152	1.536	0.099^**^	2.082	−0.043	0.349
INFR → SAT	0.082	0.769	−0.013	0.406	0.123	1.527
HOT.SER → SAT	−0.067	0.519	0.067^*^	1.809	−0.071	0.546
LEIS → SAT	0.219^**^	2.071	−0.004	0.094	0.078	0.622
CULT → SAT	0.448^***^	3.717	0.099^**^	2.103	0.196	1.570
POL.ECO → SAT	0.050	0.519	−0.010	0.359	0.089	1.025
ENVI → SAT	0.012	0.096	0.031	0.576	0.227^**^	2.117
SOCI → SAT	−0.101	1.155	−0.001	0.036	0.070	0.773
AFFE → SAT	0.392^***^	2.800	0.090^***^	2.992	0.456^***^	3.431
	*R*^2^ Sat = 0.703		*R*^2^ Sat = 0.694		*R*^2^ Sat = 0.801	
	*R*^2^ I-Com = 0.531		*R*^2^ I-Com = 0.654		*R*^2^ I-Com = 0.777	

*** *p < 0.01*;

** *p < 0.05*;

* *p < 0.10*.

With respect to the relationship between satisfaction and future behavioral intentions, the results show a significant effect of 99% (See Table 6), which allows us to support Hypothesis 4: i.e., the satisfaction derived from browsing the Webs positively affects behavioral intention.

**Table 6:** **Structural relationship between satisfaction and behavioral intentions**.

**Relationship**	**CASTILLA-LA MANCHA 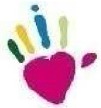 **		**Andalusia 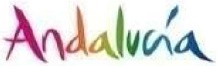 **		**CASTILLA Y LEON 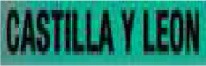 **	
	**β standard**	***t*-value (*bootst*)**	**β Standard**	***t*-value (*bootst*)**	**β standard**	***t*-value (*bootst*)**
SAT → FUT.BEH	0.729^***^	10.539	0.741^***^	15.243	0.882^***^	26.782

*** *p < 0.01*.

Finally, in order to determine whether the online information search affects the tourist's previous image, a Parametric Analysis model is developed, which allows us to determine whether significant differences exist in the strength of the relationships which might show the presence of any moderating effect (Tsang, 2002). The test makes use of the path coefficients obtained in the structural model and the standard errors of each sample in order to determine the existence of significance in the differences between said parameters, according to the different groups considered. The moderating effect which belonging to a specific group is analyzed using the *t*-test. In this regard, the results obtained (See Table 7) enable us to observe certain differences between the structural coefficients prior to browsing the tourist Web pages and those determined after the information search. Specifically, the affective image shows significant differences in the case of Castilla-La Mancha and Andalusia, while only the Web page of Castilla-La Mancha displays differences in the dimensions of the cognitive image. This allows us to confirm only partially the hypotheses H2, H2a, and H2b.

**Table 7:** **Valuation of differences**.

		**Previous**		**Subsequent**		***t***	***P-value***
		**β Standard**	**Error**	**β Standard**	**Error**		
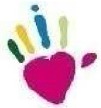	NAT.RES → SAT	−0.1237	0.1166	0.1524	0.0993	**1.818**	**0.072**
	INFR → SAT	0.1736	0.1278	0.0822	0.1068	0.553	0.581
	HOT.SER → SAT	0.2138	0.1311	−0.0672	0.1295	1.538	0.127
	LEIS → SAT	−0.1091	0.1218	0.2190	0.1057	**2.052**	**0.042**
	CULT → SAT	0.5404	0.1561	0.4483	0.1207	0.471	0.639
	POL.ECO → SAT	−0.0462	0.1058	0.0497	0.0960	0.677	0.500
	ENVI → SAT	0.2495	0.2234	0.0125	0.1308	0.923	0.358
	SOCI → SAT	−0.0563	0.0999	−0.1011	0.0875	0.340	0.734
	AFFE → SAT	0.0922	0.1303	0.3916	0.1041	1.811	0.073
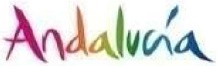	NAT.RES → SAT	0.2701	0.1126	0.0994	0.1453	0.936	0.350
	INFR → SAT	−0.1028	0.0934	−0.0129	0.1033	0.651	0.516
	HOT.SER → SAT	0.0066	0.0834	0.0666	0.1450	0.362	0.718
	LEIS → SAT	0.0368	0.0916	−0.0044	0.1276	0.264	0.792
	CULT → SAT	0.2671	0.1120	0.0987	0.1329	0.977	0.330
	POL.ECO → SAT	0.1026	0.0799	−0.0098	0.1025	0.872	0.385
	ENVI → SAT	0.0633	0.1249	0.0315	0.1185	0.186	0.852
	SOCI → SAT	0.131	0.1142	−0.0012	0.1187	0.809	0.420
	AFFE → SAT	0.3994	0.1154	0.0896	0.1029	2.021	0.045
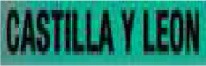	NAT.RES → SAT	−0.0879	0.1207	−0.0433	0.1243	0.260	0.796
	INFR → SAT	−0.0354	0.1062	0.1229	0.0805	1.198	0.233
	HOT.SER → SAT	0.2481	0.1255	−0.0711	0.1302	1.780	0.078
	LEIS → SAT	0.0849	0.1140	0.0778	0.1252	0.042	0.966
	CULT → SAT	0.0362	0.1166	0.1957	0.1246	0.943	0.348
	POL.ECO → SAT	−0.1217	0.1289	0.0892	0.0870	1.368	0.174
	ENVI → SAT	0.2609	0.1145	0.2267	0.1071	0.220	0.826
	SOCI → SAT	0.1279	0.1087	0.0705	0.0912	0.408	0.684
	AFFE → SAT	0.467	0.1194	0.4562	0.1330	0.061	0.952

## Conclusions and management recommendations

Information, communication technologies, and tourism comprise the services which will orientate the economy of the twenty first century. These three areas are the key to the revitalization and innovation as well as enabling the growth of Autonomous Communities in order to communicate with each other and interact with the environment. Likewise, they are tools that offer strategic opportunities and economic growth. Nevertheless, they also represent new challenges and threats for those agents, which lack the development necessary to adapt to the new advances. Information and communication technologies encourage globalization and the spread of tourism, but in turn, a demand for goods and services is generated which must be satisfied through technological progress.

Specifically, Internet has brought about changes in the direction of traditional marketing activities, given that it constitutes the ideal communication platform for a destination to offer what the different customers want and to communicate with them in different areas. Understanding how tourists acquire knowledge is important for marketing management decisions, designing effective communication campaigns, and service delivery (Srinivasan, 1990). It is during information acquisition that marketers can influence consumers' buying decisions (Schmidt and Spreng, 1996). The Internet facilitates interactive, one-to-one communication, something impossible using more traditional channels. With regard to this, in the present study an analysis has been developed regarding the effect of web pages as a communication instrument on the previous image which tourists' have of a specific destination. The assessment of destination image can assist managers by identifying the strengths and weaknesses of their destination, providing critical insights for managing and developing tourist destinations (Fakeye and Crompton, 1991; Bigne et al., 2001).

In general, the results obtained allow us to identify a set of discrepancies in the influence of promotional tourist web pages on the previous tourist image, recognizing the modifying effect of the type of destination. In the first place, differences have been identified in the final composition of the image according to the web page analyzed. The findings show that tourists determine their image of the destination based on the set of resources and attractions of the destination, which may be different to other similar destinations. These differences also mean unequal influences on the satisfaction of the tourist. Although the affective image shows a significant influence on the three destinations analyzed, only some of the dimensions of the cognitive image attain significant values in their influence on satisfaction. As in previous studies, a significant effect in the relationship between satisfaction and future behavioral intentions has been found for the three webs analyzed, which means that a tourist satisfied with the online information search has a greater likelihood of visiting the destination and recommending it to other people.

Finally, the differences have been analyzed between the tourist's previous image and the image subsequent to browsing the web page of the tourist destination. The results obtained show that only certain dimensions of the image, and only for some destinations, will the online information search modify the tourist's previous image. The reasons for the lack of significance in the effect of the web on the image may be various, such as the fact that individuals have an image of the destination so fixed that new information does not give them new data or because the web fails when it comes to faithfully and attractively transmitting the resources and capacities which characterize it. In general, the results obtained enable us to partially confirm the claims of Li et al. (2009), which conclude that the affective image is modified after browsing the Net, with the cognitive image remaining stable. In the present study it has been possible to build on these results and conclude that in the study of the relationship between the information search in the web and the modification of the image the type of destination must be considered, given that the for some destinations the information provided in their webs may mean that the affective image remains invariable, while the cognitive image is modified.

In a recent study, Fernández-Cavia et al. (2016) showed that online communications of tourist destinations are not fully professionalized and standardized, and that DMOs do not use online tools strategically, but tactically. Thus, based on this study and the results obtained, we would emphasize a series of implications which would allow destination managers to achieve better knowledge of the tourists and to develop more efficient communication strategies and promotion. In the first place, to achieve a competitive advantage through the Internet, the destinations have to ensure that their tourist resources are presented in their webs attractively and accurately, focusing on promoting those resources which allow them to improve the destination image and help it to present a better tourist experience.

Given that image depends on the destination itself, it is important to identify the tourist's previous image as well as their needs and desires, in order to develop more efficient marketing strategies. On the other hand, the destinations must be aware of the power of Internet and its potential as a communication tool. Consumers are more and more moving away from the more traditional channels, at the same as they demand a greater control over the sources of information (Vollmer and Precourt, 2008). In the paradigm of the new communications, the nature and power of the social networks must be recognized, given that consumers show greater trust in these sources of social and interactive information (Foux, 2006) and, therefore, they have a greater effect on all aspects of consumer behavior. Thus, a change of attitude in the destination managers is required who have to accept the reality of the transmission of information between consumers and, instead of speaking *to* them, must learn to converse with their consumers and in this way influence the discussions which take place in virtual spaces.

The research carried out here also presents some limitations. On the one hand, selection of the image measurements has been determined by the inclusion of aspects common to any destination considered. However, each destination possesses a set of particular characteristics and resources which in many cases may have a decisive influence on overall perception. Likewise, the selection of the web pages has followed criteria of variety as regards what the tourist is offered, although it is possible to identify other destinations that possess resources and capacities which generate a different image in the mind of the tourists. These limitations prompt us to recommend future studies such as the application of the model to other domestic destinations, including those resources and attractions typical of the destination. Second, although the relationships posited are supported by the literature, some relationships have not been included, such as the direct effect of the image on behavioral intentions, or variables which may have an influence on the attitude and behavior of the tourist, such as motivation. Therefore, we propose the inclusion of new variables and new relationships between the constructs. Finally, the selection of the sample, due to reasons of convenience, is characterized by a spatial limitation, and we would propose, as a future line of research, the enlargement of the field of investigation by using online surveys.

## Ethics statement

As far as ethical protocol, (1) we gave participants detailed and written information about the study and the procedure; (2) we refrained from collecting data related (direct or indirectly) to the subject's health and did not refer to the Declaration of Helsinki when informing the subjects; and (3) we guaranteed the anonymity of the data collected. We did not obtain prior approval from a board or committee. Because the questionnaire was completely voluntary, we took its completion as participants' consent to their data being used in our research.

## Author contributions

All authors listed, have made substantial, direct and intellectual contribution to the work, and approved it for publication.

## Funding

This work has been funded by The Ministry of Economy and Competitivity (Spain), Resarch Project with reference: ECO2014-59688-R, Programa Estatal de Investigación, Desarrollo e Innovación Orientada a los Retos de la Sociedad, Plan Estatal de Investigación Científica y Técnica y de Innovación 2013-2016, and Ayudas a Grupos de Investigación de la Universidad de Castilla-La Mancha, Orgánica 01110G6032-GI20163426.

### Conflict of interest statement

The authors declare that the research was conducted in the absence of any commercial or financial relationships that could be construed as a potential conflict of interest.

## Footnotes

^1^*Bootstrapping* (or *bootstrap*) (Chin, 1998) involves the generation of a certain number of samples with the same size as the original simple, by means of the substitution of values, obtaining the distribution of values for the standard error.
